# Genome-wide identification, molecular cloning, expression profiling and posttranscriptional regulation analysis of the *Argonaute* gene family in *Salvia miltiorrhiza*, an emerging model medicinal plant

**DOI:** 10.1186/1471-2164-14-512

**Published:** 2013-07-29

**Authors:** Fenjuan Shao, Shanfa Lu

**Affiliations:** 1 Institute of Medicinal Plant Development, Chinese Academy of Medical Sciences & Peking Union Medical College, No.151, Malianwa North Road, Haidian District, Beijing 100193, China

## Abstract

**Background:**

Argonaute (AGO) is the core component of RNA-induced silencing complex. The *AGO* gene family has been analyzed in various plant species; however, there is no report about *AGOs* in the well-known Traditional Chinese Medicine (TCM) plant, *Salvia miltiorrhiza*.

**Results:**

Through a genome-wide analysis, we identified ten *SmAGO* genes in *S. miltiorrhiza*. Full-length cDNAs of all *SmAGOs* were subsequently cloned and sequenced. These *SmAGOs* were characterized using a comprehensive approach. Sequence features, gene structures and conserved domains were analyzed by the comparison of *SmAGOs* and *AtAGOs*. Phylogenetic relationships among AGO proteins from *S. miltiorrhiza*, *Arabidopsis* and rice were revealed. The expression levels of *SmAGO* genes in various tissues of *S. miltiorrhiza* were investigated. The results implied that some *SmAGOs*, such as *SmAGO1*, *SmAGO2*, *SmAGO3*, *SmAGO7* and *SmAGO10*, probably played similar roles as their counterparts in *Arabidopsis*; whereas the others could be more species-specialized. It suggests the conservation and diversity of *AGOs* in plants. Additionally, we identified a total of 24 hairpin structures, representing six miRNA gene families, to be miRNA precursors. Using the modified 5′-RACE method, we confirmed that *SmAGO1* and *SmAGO2* were targeted by *S. miltiorrhiza* miR168a/b and miR403, respectively. It suggests the conservation of AGO1-miR168 and AGO2-miR403 regulatory modules in *S. miltiorrhiza* and *Arabidopsis*.

**Conclusions:**

This is the first attempt to explore *SmAGOs* and miRNAs in *S. miltiorrhiza*. The results provide useful information for further elucidation of gene silencing pathways in *S. miltiorrhiza*.

## Background

Small RNAs (sRNAs) involved in various gene silencing pathways play important and diverse roles in the development and differentiation of organisms through regulating gene expression at the transcriptional and post-transcriptional levels [1], affecting heterochromatin formation [2], and responding to biotic and abiotic stresses [3]. In plants, sRNAs are generated from double-stranded RNAs (dsRNAs) through various pathways and may be classified into two major classes, including microRNAs (miRNAs) and small interfering RNAs (siRNAs), based on the source of dsRNA [4]. miRNAs are produced from plant transcripts with internal stem-loop structures, whereas siRNAs are derived from transcripts with inverted-repeat sequence, dsRNAs copied from single-stranded RNA (ssRNA), over-lapping regions of bidirectional transcripts, or dsRNAs formed by virus replication. The biogenesis pathways of plant sRNAs involve in various gene families, such as the Dicer-like (*DCL*) family and the RNA dependent RNA polymerase (*RDR*) family, and each pathway appears to be taken part in by different member of a gene family [4]. To regulate gene expression, the generated sRNA duplexes from dsRNAs are loaded into RNA-induced silencing complexes (RISCs) with Argonautes (AGOs) as the central components [5]. RISCs remove the star strand (known as miRNA* or siRNA*) of sRNA duplex and select the functional strand as a guide to interact with homologous RNA or DNA molecules for direct RNA cleavage, translational repression or DNA methylation [6,7].

AGOs are ribonucleases with two conserved domains, including PAZ and PIWI [8]. The PAZ domain contains a specific binding pocket that can anchor sRNA duplexes with two-nucleotide 3′ overhang. The PIWI domain exhibits endonuclease activity and the structure of PIWI domain folded is similar to RNase H [9]. The endonuclease activity of PIWI domain is performed by an active site usually carrying an Asp–Asp–His (DDH) or Asp–Asp–Asp (DDD) motif. AGOs are usually encoded by a multiple gene family in organisms and the number of *AGO* genes differs in different organisms [10]. Fission yeast has only one *AGO* gene, whereas insects, mammals and worms have five, eight, and twenty six *AGO* genes, respectively. Annotation of the *Arabidopsis* and rice genomes revealed ten and eighteen *AGO* genes, respectively [11]. Additionally, a total of eighteen maize and fifteen tomato *AGO* genes have been identified [12,13]. Although a large number of *AGO* genes have been found in various plants, the majority were predicted by computational approaches based on sequence similarity. Only a small proportion of known *AGOs* were identified or confirmed by full-length cDNA cloning. Among them, *Arabidopsis AGO1* is the most well-studied plant *AGO* gene. It encodes the core component of RISCs associated with the action of miRNAs, trans-acting siRNAs (ta-siRNAs) and transgene-derived siRNAs [14]. AGO2 protein is involved in antiviral defense by catalyzing viral RNA cleavage in *Arabidopsis* plants [15]. *Arabidopsis AGO4*, *AGO6* and *AGO9* genes recruit endogenous 24nt sRNAs for DNA methylation, which causes target gene silencing at the transcriptional level [16,17]. *AGO7* is involved in the generation of ta-siRNAs from *TAS3* by collaborating with miR390 in *Arabidopsis*[18]. *Arabidopsis AGO10* gene modulates shoot apical meristem maintenance and establishment of leaf polarity by repressing miR165/166 [19,20]. In addition, although AGO proteins are central components of RISCs involved in sRNA-mediated RNA cleavage, translational repression and DNA methylation, some of them are regulated by sRNAs through the feedback mechanism. For instance, the expression level of *AtAGO1* is regulated by miR168 through direct cleavage of *AtAGO1* transcripts [21]. Similarly, *AtAGO2* is regulated by miR403 [22]. The function and regulatory mechanism of *AGO* genes from plant species other than *Arabidopsis* is largely unknown.

*S. miltiorrhiza*, which produces two major groups of bioactive compounds, lipophilic diterpenoid tanshinones and hydrophilic phenolic acids, is a well-known traditional Chinese medicine (TCM) widely used for treating various human diseases, such as dysmenorrhoea, amenorrhoea and cardiovascular disease, for thousands of years [23,24]. It is also an emerging model plant for TCM studies because of its relatively small genome size, short life cycle, undemanding growth requirements, and significant medicinal value [25,26]. The *S. miltiorrhiza* genome has been preliminarily decoded and a working draft of the genome is currently available (Chen et al., unpublished data). The interest of gene silencing pathways in *S. miltiorrhiza* is increasing. With the aim to elucidate the core components of gene silencing pathways, we performed a genome-wide prediction of the *S. miltiorrhiza AGO* gene family. Molecular cloning of *AGO* genes was carried out for validation and error correction of computational prediction. The characteristics of *S. miltiorrhiza AGOs* were revealed by a comprehensive analysis, including comparison with *AGOs* from other plant species, gene expression profiling, and analysis of posttranscriptional regulation. The results provide useful information for further elucidation of gene silencing pathways in *S. miltiorrhiza*.

## Results

### Genome-wide prediction of *S. miltiorrhiza AGO* genes

To predict *S. miltiorrhiza AGO* genes at the genome level, we downloaded all of 10 *Arabidopsis* and 19 rice AGO amino acid sequences from GenBank (http://www.ncbi.nlm.nih.gov/protein). BLAST analysis of *Arabidopsis* and rice AGOs against the current assembly of the *S. miltiorrhiza* genome (Chen et al., unpublished) was then performed using the tBLASTn algorithm [27]. An e-value cut-off of 10^-10^ was applied to the homologue recognition. As a result, a total of 10 genomic loci of *SmAGO* genes were identified. The genomic DNA sequence was retrieved and gene models of 10 *SmAGO* genes were predicted using Genscan (http://genes.mit.edu/GENSCAN.html) [28]. The models were further examined and corrected manually by comparison with *AGO* genes identified from other plant species using the BLASTx algorithm (http://www.ncbi.nlm.nih.gov/BLAST) [27]. All of 10 deduced proteins share high sequence similarity with known plant AGOs and contain the conserved PAZ and PIWI domains, suggesting they are authentic AGOs. The identified *AGO* genes are named *SmAGO1* to *SmAGO10*, respectively, based on high sequence similarity with corresponding *Arabidopsis AGOs*.

### Sequence feature, gene structure and conserved domain comparison of *SmAGOs* and *AtAGOs*

**Figure 1:**
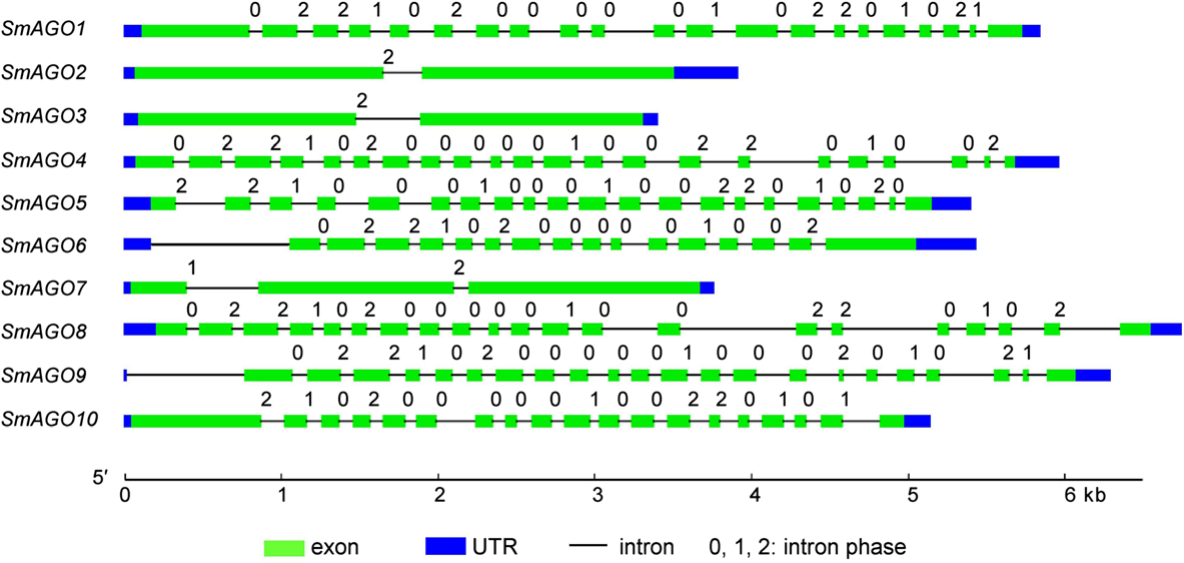
**Gene structures of *AGOs *in *S. miltiorrhiza*.** Exons, UTRs, introns and intron phases are shown.

**Figure 2:**
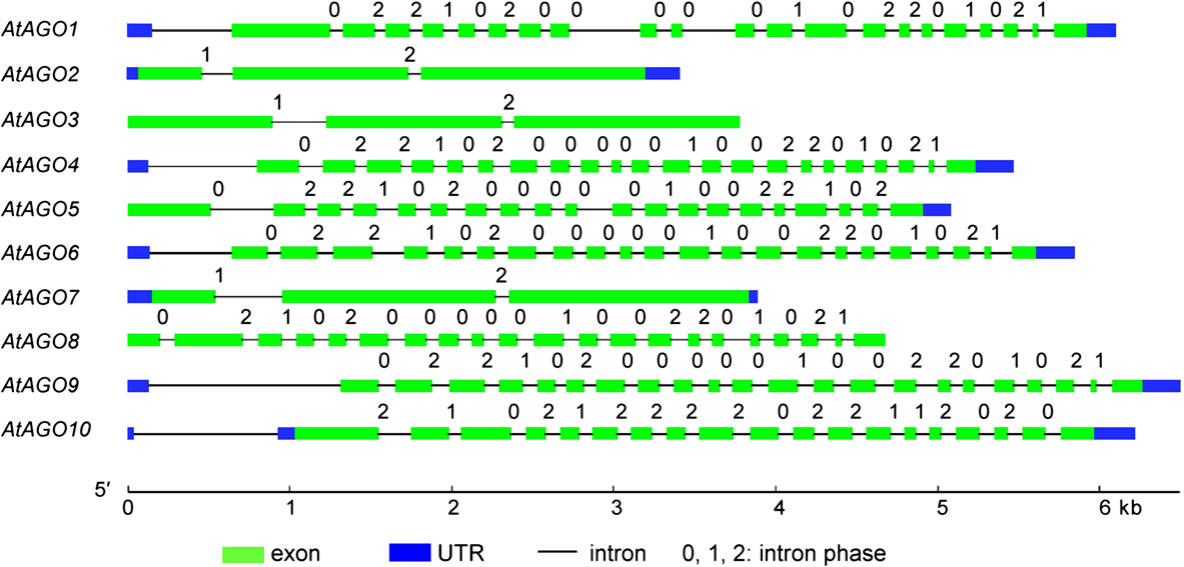
**Gene structures of *AGOs *in *Arabidopsis*.** Exons, UTRs, introns and intron phases are shown.

It is very important to know the correct cDNA sequence for systematic characterization of AGOs in *S. miltiorrhiza*. In order to confirm the results from prediction and correct errors of computation, molecular cloning of full-length *SmAGO* cDNA was carried out. As a result, all of ten predicted *SmAGO* genes were experimentally validated. Analysis of sequence features showed that the length of open reading frames (ORFs) of *SmAGOs* varied from 2,472 (*SmAGO5*) to 3,195 bp (*SmAGO1*) (Table 1). The length of 5′ and 3′ UTRs was between 30 and 202 bp and between 91 and 337 bp, respectively. The size of deduced SmAGO proteins varied between 823 and 1064 amino acids, the molecular weight (Mw) varied from 93.2 to 118.7 kDa, and the theoretical p*I* was between 9 and 10 (Table 1). These sequence features are quite similar to that of *AtAGOs* in *Arabidopsis* (Table 2). The similarity between *SmAGOs* and *AtAGOs* was also observed in gene structures. Many *SmAGOs* genes have the number and phase of introns similar to an *AtAGO*, such as *SmAGO1*/*AtAGO1*, *SmAGO4*/*AtAGO4*, *SmAGO7*/*AtAGO7*, *SmAGO8*/*AtAGO8*, and *SmAGO9*/*AtAGO9* (Tables 1 and 2, Figures 1 and 2). Additionally, both *SmAGO6*/*AtAGO6* and *SmAGO9*/*AtAGO9* have an intron in 3′ UTR (Figures 1 and 2). The results suggest the conservation between *SmAGOs* and *AtAGOs*.

**Table 1:** **Sequence features and intron number of *SmAGOs***

**Gene name**	**Accession number**	**cDNA (bp)**	**ORF (bp)**	**5′UTR (bp)**	**3′UTR (bp)**	**Protein (aa)**	**Mw (kDa)**	**p*I***	**Intron no.**
*SmAGO1*	KF153679	3429	3195	121	113	1064	118.7	9.68	20
*SmAGO2*	KF153680	3369	2964	68	337	988	110.1	9.85	1
*SmAGO3*	KF153681	2975	2793	86	96	930	106.0	9.86	1
*SmAGO4*	KF153682	2974	2613	81	280	870	97.7	9.16	21
*SmAGO5*	KF153683	2905	2472	177	256	823	93.2	9.64	20
*SmAGO6*	KF153684	3212	2712	172	328	903	101.1	9.37	12
*SmAGO7*	KF153685	3182	3045	46	91	1014	115.5	9.28	2
*SmAGO8*	KF153686	3075	2673	202	200	890	100.0	9.79	18
*SmAGO9*	KF153687	2915	2667	30	218	888	98.7	9.24	21
*SmAGO10*	KF153688	3118	2907	39	172	968	108.9	9.44	18

**Table 2:** **Sequence features and intron number of *AtAGOs***

**Gene name**	**Gene model**	**cDNA (bp)**	**ORF (bp)**	**5′UTR (bp)**	**3′UTR (bp)**	**Protein (aa)**	**Mw (kDa)**	**p*I***	**Intron no.**
*AtAGO1*	AT1G48410.1	3601	3147	267	187	1048	116.2	9.65	21
*AtAGO2*	AT1G31280.1	3343	3045	66	232	1014	113.4	9.97	2
*AtAGO3*	AT1G31290.1	3585	3585	-	-	1194	129.2	9.78	2
*AtAGO4*	AT2G27040.1	3170	2775	134	261	924	102.9	9.19	22
*AtAGO5*	AT2G27880.1	2994	2994	-	-	997	111.1	9.92	19
*AtAGO6*	AT2G32940.1	2997	2637	139	221	878	98.7	8.59	22
*AtAGO7*	AT1G69440.1	3160	2973	150	37	990	113.4	9.59	2
*AtAGO8*	AT5G21030.1	2553	2553	-	-	850	95.5	8.92	20
*AtAGO9*	AT5G21150.1	3017	2691	115	211	896	100.5	9.45	22
*AtAGO10*	AT5G43810.1	3330	2967	128	235	988	110.9	9.64	18

Search *S. miltiorrhiza* AGO proteins for conserved domains against the NCBI Conserved Domain Database (CCD) revealed that all SmAGOs contained DUF1785, PAZ, and PIWI domains (see Additional file 1), which were consistent with the results from other plant AGOs [29]. The function of DUF1785 is currently unknown; whereas PAZ has been shown to play roles in binding sRNA duplexes and PIWI are important in cleavage of target RNA [30,31]. The endonuclease activity of PIWI domain is performed by an active site usually carrying a DDH or DDD motif [15,32]. Additionally, a conserved histidine at position 798 of *Arabidopsis* AGO1 was found to be critical for *in vitro* endonuclease activity [14]. Analysis of ten *S. miltiorrhiza* AGOs showed that half of them, namely SmAGO1, SmAGO2, SmAGO3, SmAGO7 and SmAGO10, contained the conserved DDH/H798 or DDD/H798 residues; whereas the conserved residues were not observed in the other SmAGOs (see Additional file 2). In SmAGO4, SmAGO5 and SmAGO8, the third histidine was missing or replaced by leucine. SmAGO6 and SmAGO9 possessed the conserved DDH triad but histidine at 798th position was either replaced by alanine or proline (see Additional file 2).

### Phylogenetic analysis of AGO proteins in *S. miltiorrhiza*, *Arabidopsis* and rice

**Figure 3:**
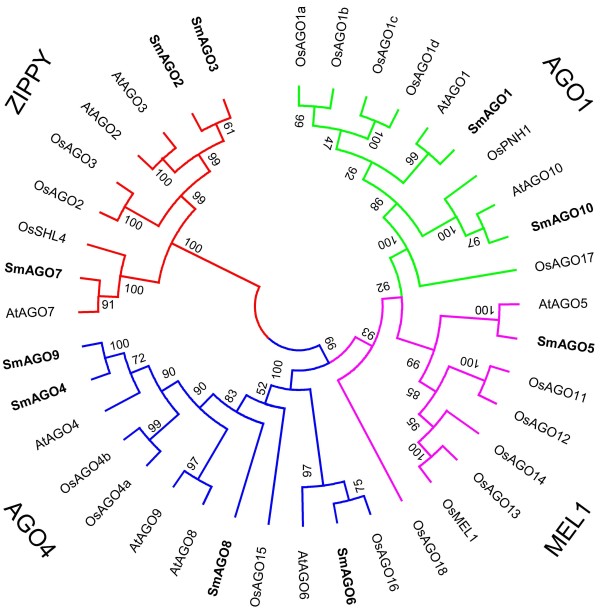
**Unrooted neighbor-joining phylogenetic tree of AGOs from *S. miltiorrhiza*, *Arabidopsis *and rice.** The deduced full-length amino acid sequences were aligned using ClustalW version 1.83 and the phylogenetic tree was constructed using MEGA 4.0 by the neighbor-joining (NJ) method with 1000 bootstrap replicates. Four subgroups, including AGO1, MEL1, AGO4 and ZIPPY, are indicated. SmAGOs are shown in bold.

Phylogenetic analysis using the PAZ and PIWI domains for rice, *Arabidopsis*, *Caenorhabditis elegans*, *Drosophila melanogaster*, and mouse had previously revealed that animal AGOs clustered into two subgroups: A1 and A2, whereas all plant AGOs could be divided into four subgroups: AGO1, ZIPPY, AGO4, and MEL1 [33]. The AGO1 and MEL1 subgroups had a common lineage with A1, whereas plant ZIPPY and AGO4 subgroups and animal A2 subgroup could be diverged from an ancestral lineage [11]. In order to determine the evolutionary relationship of *S. miltiorrhiza* AGOs, full-length AGO protein sequences from *S. miltiorrhiza*, *Arabidopsis* and rice were aligned and an unrooted neighbor-joining tree was constructed. The results showed that ten SmAGOs could also be divided into four subgroups (Figure 3). Moreover, the clustering remained similar when only the PAZ and PIWI protein domains were used for phylogenetic analysis (data not shown). SmAGO1 and SmAGO10 are included in the AGO1 subgroup with *Arabidopsis* AtAGO1 and AtAGO10, and rice OsAGO1a-OsAGO1d and OsPNH1. SmAGO1 shares high similarity with AtAGO1 associated with the action of miRNAs, ta-siRNAs and transgene-derived siRNAs [14], whereas SmAGO10 is highly similar to AtAGO10 modulating shoot apical meristem maintenance and establishment of leaf polarity [20]. SmAGO2, SmAGO3 and SmAGO7 are members of the subgroup ZIPPY. Similarly, three *Arabidopsis* AGOs, including antiviral defense-associated AtAGO2, function-unknown AtAGO3 and AtAGO7 involved in the generation of ta-siRNAs from *TAS3*[15], are also included in this subgroup. SmAGO7 shares greater similarity with AtAGO7. SmAGO2 and SmAGO3 are highly similar to AtAGO2 and AtAGO3. The MEL1 subgroup contains only one *S. miltiorrhiza* AGO, SmAGO5, which is similar to the function-unknown *Arabidopsis* AtAGO5. On the contrary, the AGO4 subgroup is the biggest among four plant AGO subgroups. It contains five *S. miltiorrhiza* AGOs, including SmAGO4, SmAGO6, SmAGO8 and SmAGO9.

### Differential expression of *SmAGO* genes

**Figure 4:**
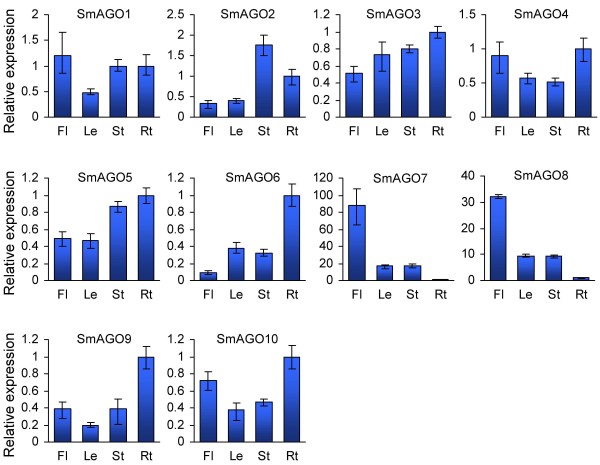
**Expression of *SmAGOs *in flowers (Fl), leaves (Le), stems (St) and roots (Rt) of *S. miltiorrhiza*.** Fold changes of *SmAGO* expression are shown. Expression levels were quantified by qRT-PCR. *SmUBQ10* was used as a reference gene. The levels in roots were arbitrarily set to 1. Error bars represent the standard deviations of three technical PCR replicates.

The expression of *SmAGO* genes in flowers, leaves, stems and roots of 2-year-old, field nursery-grown *S. miltiorrhiza* was analyzed using quantitative RT-PCR technology. *SmUBQ10* was chosen as an endogenous control as previously described [26]. All of ten *SmAGOs* were expressed in *S. miltiorrhiza* tissues analyzed, whereas differential expression patterns were observed (Figure 4). *SmAGO1* and *SmAGO10* clustered in the AGO1 subgroup were expressed in flowers, leaves, stems and roots as their *Arabidopsis* counterparts, *AtAGO1* and *AtAGO10*, respectively [11]. It is consistent with the ubiquitous roles of *AGOs* in the AGO1 subgroup. Although *SmAGO2*, *SmAGO3* and *SmAGO7* were clustered in the ZIPPY subgroup, their expression patterns were distinct (Figure 4). On the other hand, the expression patterns of *SmAGO2*, *SmAGO3* and *SmAGO7* were quite similar to their *Arabidopsis* counterparts, *AtAGO3*, *AtAGO2*, and *AtAGO7*, respectively [11]. By contrast, the expression pattern of *SmAGO5* was distinct with their *Arabidopsis* and rice counterparts in the MEL1 subgroup. *SmAGO5* showed the highest expression in roots, followed by stems, less in flowers and leaves. It is distinct with its counterparts, *AtAGO5* in *Arabidopsis* and *OsMEL1*, *OsAGO12*, *OsAGO13* and *OsAGO14* in rice, showing specifically expression in reproductive tissues (Figure 4) [34]. Similar to the *SmAGOs* in the ZIPPY subgroup, *SmAGO4*, *SmAGO6*, *SmAGO8* and *SmAGO9* clustered in the AGO4 subgroup exhibited divergent expression patterns (Figure 4). *SmAGO8* was more flower-specific, an expression pattern similar with *SmAGO7* in the ZIPPY subgroup; whereas, *SmAGO6* and *SmAGO9* were more root-specific. The results indicate the functional conservation and diversity of *AGOs*.

### miRNA-mediated posttranscriptional regulation of *SmAGO* genes

**Figure 5:**
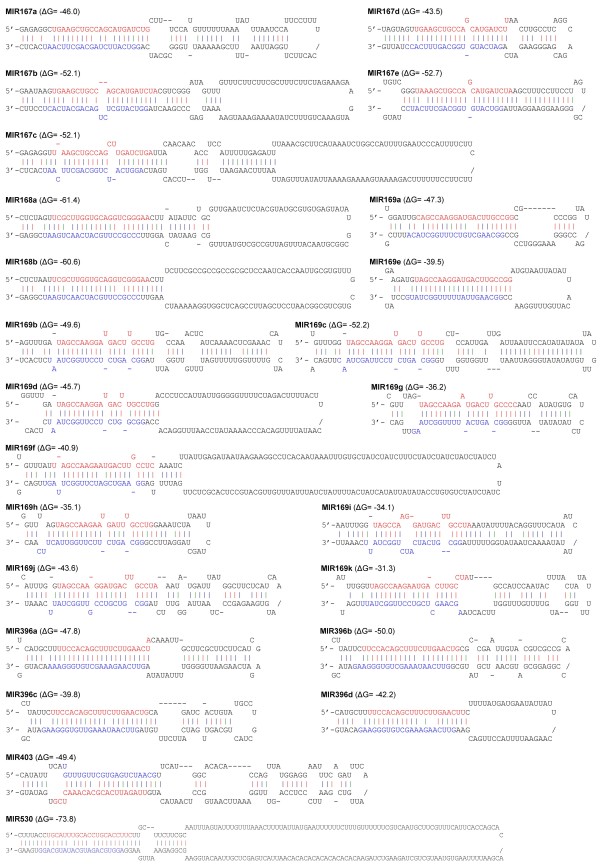
**Predicted hairpin structures of *S. miltiorrhiza *miRNA precursors.** Mature miRNA sequences are indicated in red. miRNA* are indicated in blue. The red, green and blue vertical lines indicate G:C, G:U and A:U pairing, respectively.

**Figure 6:**
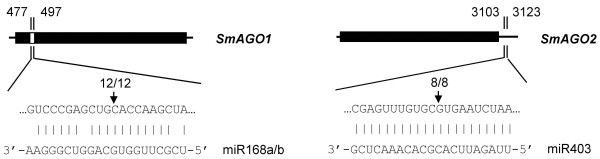
**Experimental validation of miR168a/b- and miR403-mediated cleavage of *SmAGO1 *and *SmAGO2*, respectively.** Cleavage sites were determined by the modified 5′ RNA ligase-mediated RACE. Heavy black lines represent ORFs. The lines flanking gray regions represent nontranslated regions. miRNA complementary sites with the nucleotide positions of *SmAGO1* and *SmAGO2* cDNAs are indicated. The RNA sequence of each complementary site from 5′ to 3′ and the predicted miRNA sequence from 3′ to 5′ are shown in the expanded regions. Watson-Crick pairing is indicated by vertical dashes. Vertical arrows indicate the 5′ termini of miRNA-guided cleavage products, as identified by 5′-RACE, with the frequency of clones shown.

**Figure 7:**
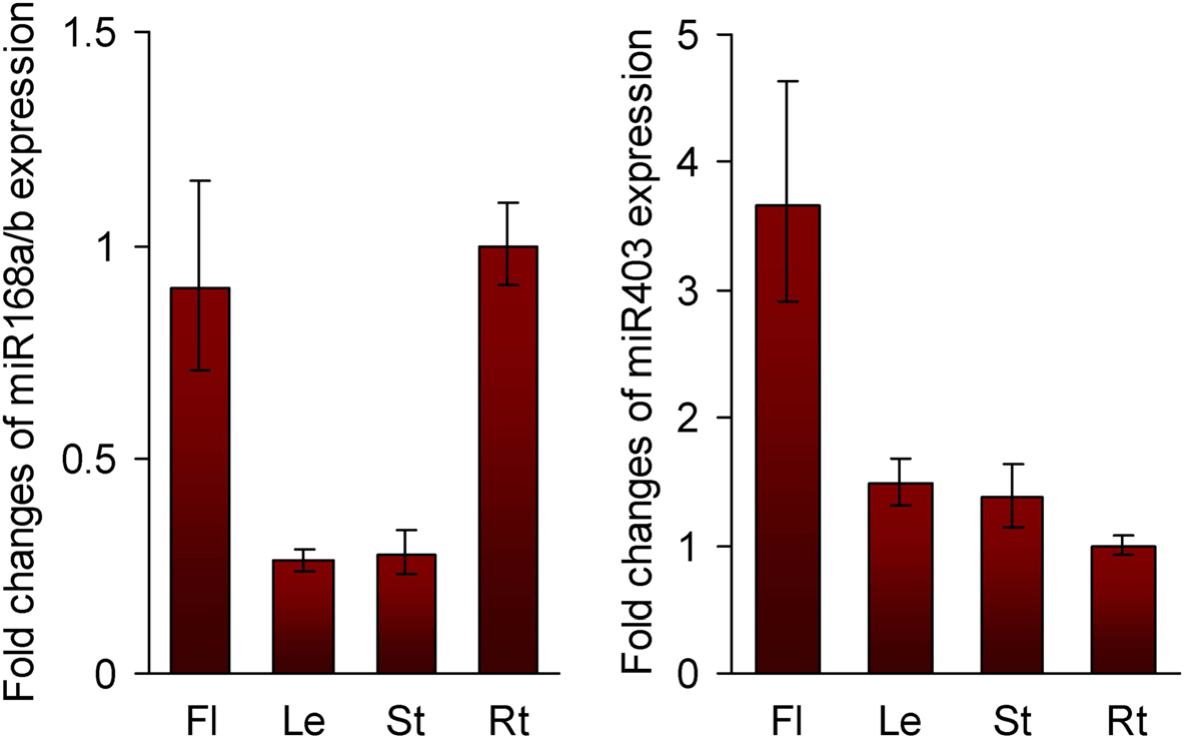
**Expression of miR168a/b and miR403 in flowers (Fl), leaves (Le), stems (St) and roots (Rt) of *S. miltiorrhiza*.** Fold changes of miRNA expression are shown. Expression levels were quantified by miRNA-specific qRT-PCR. *S. miltiorrhiza* 5.8S rRNA was used as a reference gene. The levels in roots were arbitrarily set to 1. Error bars represent the standard deviations of three technical PCR replicates.

To determine whether *S. miltiorrhiza AGOs* are regulated by miRNAs, we performed a target search of plant miRNAs in miRBase against ten full-length *SmAGO* cDNA sequences using psRNATarget [35,36]. The maximum expectation of 3.5 was applied in the target search. A total of 31 miRNA families were found to have perfect or near-perfect complementarity to *SmAGOs*. It includes 28 mature miRNAs and 3 likely miRNA* (aly-miR167d-3p, ptc-miR169n-3p, and gma-miR396j). Plant miRNA sequences belonging to the 31 families were aligned with the current assembly of the *S. miltiorrhiza* genome using SOAP2 with two mismatches allowed [37]. Genomic DNA fragments surrounding these miRNA sequences were used to predict the secondary structure using the mfold program [38]. A total of 24 hairpin structures were identified for six miRNA families, including miR167, miR168, miR169, miR396, miR403 and miR530 (Figure 5). Manual examination of the complementarities between *SmAGOs* and the identified *S. miltiorrhiza* miRNAs/miRNAs* and calculation of penalty scores as previously described [39] showed that the scores for *S. miltiorrhiza* miR168:*SmAGO1*, miR403:*SmAGO2* and miR530:*SmAGO1* were 2, 0, and 3.5, respectively. However, the scores for miR167*:*SmAGO8*, miR169*:*SmAGO5*, miR396*:*SmAGO3* and were at least 5.5, 9.5, and 5.0, respectively. High penalty scores for *S. miltiorrhiza* miRNA*:*SmAGOs* were due to low conservation among plant miRNAs*. To verify whether these miRNAs can mediate the cleavage of *SmAGO* transcripts, we isolated RNAs from roots and pooled samples containing flowers, leaves, stem and roots of *S. miltiorrhiza* and performed the modified 5′-rapid amplification of cDNA ends (RACE) for SmAGOs. The 5′-RACE products revealed that *SmAGO1* and *SmAGO2* are indeed the targets of *S. miltiorrhiza* miR168 and miR403, respectively (Figure 6). miR168 targets to the DUF1785 domain (from 477 to 497 bp) region of *SmAGO1*, while the target site of miR403 is within the 3′UTR of *SmAGO2*. It is consistent with the results from *Arabidopsis*[21,22], suggesting the conservation of miR168- and miR403-mediated regulation of *AGOs* between *S. miltiorrhiza* and *Arabidopsis*. We also cloned 5′-RACE products with the 5′-end mapped to the miR169* complementary region of *SmAGO5*; however, the positions are not between the 10 and 11 nucleotides from the 5′-end of the miRNA (data not shown). No 5′-RACE products were obtained for *SmAGO8*, *SmAGO3* and *SmAGO1* that were predicted to be targeted by miR167, miR396, and miR530, respectively. In addition, miRNA-specific qRT-PCR [40] was performed to examine the expression patterns of *S. miltiorrhiza* miR168 and miR403 experimentally validated to target *SmAGO1* and *SmAGO2* for cleavage. The results showed that miR168 and miR403 were expressed in all tissues analyzed. The levels of miR168 in roots and flowers were about three times the levels in leaves and stems. The level of miR403 in flowers was more than twice the level in other tissues analyzed (Figure 7).

## Discussion

### Identification of ten full-length *AGO* cDNAs in *S. miltiorrhiza*

Although Argonautes play very important roles in small RNA-mediated gene silencing and a large number of *AGO* genes have been found in plants, many of them were identified through computational prediction based on sequence similarity. For example, often *Arabidopsis AtAGOs*, seven, including *AtAGO1*, *AtAGO2*, *AtAGO4*, *AtAGO6*, *AtAGO7*, *AtAGO9* and *AtAGO10*, have been experimentally tested, whereas the other three, including *AgAGO3*, *AtAGO5* and *AtAGO8*, were predicted computationally (http://www.arabidopsis.org/). Among nineteen rice Os*AGO* genes, only six, including *OsAGO1a*, *OsAGO1b*, *OsAGO1c*, *OsAGO1d*, *OsAGO7* and *OsPNH1*, have been cloned (http://www.ricedata.cn/gene). Except for *SlAGO1-1*, *SlAGO1-2* and *SlAGO7*, twelve of fifteen tomato *SlAGO* genes have not been experimentally confirmed [41,42]. In this study, we performed a genome-wide prediction of ten *SmAGOs* using computational approaches, and then cloned the full-length cDNAs of all predicted *SmAGOs*. The number of identified *S. miltiorrhiza AGO* genes is comparable with that in *Arabidopsis*, although it is significantly less than the number in rice and maize, which are nineteen and eighteen, respectively [11,12]. It indicates that less duplication events are occurred for *AGO* genes in *S. miltiorrhiza* and *Arabidopsis* as compared with rice and maize *AGOs*, most of which are evolved by duplication events [11,12]. The results provide very useful information for further elucidation of *AGO* functions in *S. miltiorrhiza* and gene model prediction of *AGOs* in other plant species.

### Conservation and diversity of SmAGOs and AtAGOs

Plant AGO proteins share three highly conserved domains, including DUF1785, PAZ, and PIWI [43]. Consistently, all SmAGOs were found to contain these domains (see Additional file 1). PAZ functions in binding sRNA duplexes and PIWI is involved in RNA cleavage, whereas the function of DUF1785 remains to be elucidated [30,31]. The conserved DDH/H798 or DDD/H798 residues in PIWI domain have been demonstrated to be critical for the endonuclease activity of AGO proteins [15,32]. The conserved residues were also found in five of ten SmAGOs (see Additional file 2). It includes SmAGO1 and SmAGO10 belonging to the AGO1 subgroup and SmAGO2, SmAGO3 and SmAGO7 included in the ZIPPY subgroup. Consistently, expression profiling of these *SmAGOs* revealed similar patterns with their *Arabidopsis* counterparts (Figure 4) [11]. It indicates *S. miltiorrhiza AGOs* in the AGO1 and ZIPPY subgroup may play similar functions in the action of miRNAs, ta-siRNAs and transgene-derived siRNAs and in antiviral defense as their counterparts in *Arabidopsis*[15].

On the other hand, in the other five SmAGOs, one or two of the conserved residues was missing or replaced by other residues. It includes SmAGO5 belonging to the MEL1 subgroup, and SmAGO4, SmAGO6, SmAGO8 and SmAGO9 included in the AGO4 subgroup. Comparison of AGOs revealed that many *Arabidopsis* and rice AGOs belonging to the MEL1 and AGO4 subgroups were also lack of the conserved DDH/H798 or DDD/H798 motif, such as *Arabidopsis* AtAGO4, AtAGO6, AtAGO8 and AtAGO9, rice OsAGO4a, OsAGO4b, OsAGO15 and OsAGO16 included in the AGO4 subgroup, and rice OsAGO11, OsAGO13, OsAGO14 and OsAGO18 belonging to the MEL1 subgroup [11,13]. The function of AGOs in the MEL1 subgroup is currently unknown, whereas three of four *Arabidopsis* AGOs included in the AGO4 subgroups have been shown to recruit endogenous 24nt sRNAs for DNA methylation [44-46]. It indicates that some of SmAGOs in the MEL1 and AGO4 subgroups probably play a role in DNA methylation instead of RNA cleavage in *S. miltiorrhiza*. Since the expression patterns of *SmAGOs* in the MEL1 and AGO4 subgroups are distinct with their *Arabidopsis* counterparts (Figure 4) [11], some SmAGOs in these subgroups may play more species-specialized roles. Further elucidation of these species-specialized roles will definitely add new insights into AGO-associated gene silencing. In addition, absence of the conserved DDH/H798 or DDD/H798 motif was also found in some AGO proteins from other plant species, such as maize and tomato [12,13]. It is possible that some of the AGO proteins without the deeply conserved DDH/H798 or DDD/H798 motif are still capable of target RNA cleavage. In this case, the unconserved residues in the motif may contribute to endonuclease activity. Otherwise, they may be not a cleavage component if the deletion or replacement of conserved residues in the motif results in loss of endonuclease activity. Further experimental analysis is necessary to clarify the hypothesis.

### *S. miltiorrhiza* miRNA identification and posttranscriptional regulation of *SmAGO* genes

miRNAs are a class of small endogenous non-coding RNAs with size about 21 nucleotides. They are derived from primary miRNAs (pri-miRNAs) transcribed from miRNA loci [47]. Pri-miRNAs have internal stem-loop structures that are cleaved by DCL1 to form miRNA precursors, known as pre-miRNAs. miRNAs play vital roles in plant development and stress responses and have been identified from various plant species [39,48,49]; however, there is no report for miRNAs in *S. miltiorrhiza*, an emerging model medicinal plant. Through a computational approach, we predicted 24 miRNA genes in *S. miltiorrhiza* for the first time. They represent 6 miRNA gene families, including miR167, miR168, miR169, miR396, miR403 and miR530 (Figure 5). Using the modified 5′-RACE method, we confirmed that *SmAGO1* and *SmAGO2* were targeted by *S. miltiorrhiza* miR168a/b and miR403, respectively (Figure 6). Consistently, in *Arabidopsis*, *AtAGO1* and *AtAGO2* were also regulated by miR168 and miR403, respectively [21,22]. It suggests the existence of conserved regulatory mechanism for some *AGOs* in *S. miltiorrhiza* and *Arabidopsis*.

It is generally considered that miRNA expression is negatively correlated with that of targeted mRNAs [50]. However, there are many exceptions. For instance, miR160, miR164 and miR172 showed a positive correlation with their targets in rice [51]. Comparing the expression of *S. miltiorrhiza* miRNAs and their targets showed that no simple linear correlations existed between miR168 and *SmAGO1* and between miR403 and *SmAGO2* (Figures 4 and 7). It could be a consequence of the feedback regulation of miR168/AGO1 and miR403/AGO2 [21,22]. It is also possible that these miRNAs and targets are regulated by other unidentified factors associated with a more complex regulation [52,53].

Although we cloned cDNA fragments with the 5′-end mapped to the miR169* complementary region of *SmAGO5*, they were probably not the products of miR169*-directed cleavage. First, the penalty scores for miR169*:*SmAGO5* were at least 9.5, suggesting low complementarity between miR169* and *SmAGO5*. Second, the positions mapped were not located between the 10 and 11 nucleotides from the 5′-end of miR169*, which was atypical for miRNA-directed cleavage [54].

*S. miltiorrhiza* miR167*, miR396* and miR530 were computationally predicted to target *SmAGO8*, *SmAGO3* and *SmAGO1* for cleavage, but no 5-RACE products were obtained for these *SmAGOs*. It indicates that these miRNAs are probably not involved in the regulation of *SmAGOs*. However, we can not rule out the possibility that some of the miRNAs regulate *SmAGO* mRNA levels in tissues unanalyzed or at specific developmental stages of *S. miltiorrhiza* or in cells undergoing certain environmental stresses. It is also possible that some of the miRNAs interact with *SmAGO* mRNAs for translational repression. Further analyzing the interaction between miRNAs and *SmAGOs* through genetic transformation may give us a clearer picture about the regulatory mechanism of *SmAGOs*.

## Conclusions

The decoding of *S. miltiorrhiza* genome allowed us to perform a genome-wide prediction of *SmAGO* genes. The prediction was further confirmed by full-length cDNA cloning, which resulted in identification of the first set of full-length *AGO* cDNAs in a plant species. Through a comprehensive approach, combining sequence feature, gene structure and conserved domain comparison of *SmAGOs* and *AtAGOs*; phylogenetic analysis of AGO proteins in *S. miltiorrhiza*, *Arabidopsis* and rice; and expression of *SmAGO* genes *S. miltiorrhiza*, we characterized the newly identified 10 *SmAGOs*. The results suggest the conservation and diversity of sequence features and gene functions among *AGOs* from different plant species. Analysis of posttranscriptional regulation of *AGO* genes suggested the existence of conserved AGO1-miR168 and AGO2-miR403 regulatory modules in *S. miltiorrhiza* and *Arabidopsis*. These results will help to open a window for understanding gene silencing networks in the well-known TCM plant, *S. miltiorrhiza*.

## Methods

### Plant materials

*S. miltiorrhiza* Bunge (line 993) with whole genome sequences available was grown in a field nursery. Mature flower buds blooming soon, mature and healthy leaves, young stems and roots in about 0.5 cm diameter were collected from 2-year-old plants on Aug. 15, 2012. Samples from three plants were pooled and stored in liquid nitrogen until use.

### Prediction of *SmAGO* genes

*Arabidopsis* and rice AGO protein sequences were downloaded from GenBank (http://www.ncbi.nlm.nih.gov/protein) and used to search for homologues against the current assembly of the *S. miltiorrhiza* genome (Chen et al., unpublished) using the tBLASTn algorithm [27]. An e-value cut-off of 10^-10^ was applied to the homologue recognition. All retrieved sequences were used for gene prediction on the Genscan web server (http://genes.mit.edu/GENSCAN.html) [28]. The predicted gene models were further examined and corrected manually by comparison with *AGO* genes identified from other plant species using the BLASTx algorithm (http://www.ncbi.nlm.nih.gov/BLAST) [27].

### Cloning of *SmAGO* genes

Total RNA was extracted from the root of *S. miltiorrhiza* using Trizol reagent (invitrogen). mRNA was purified using the oligotex mRNA mini kit (invitrogen). RNA ligase-mediated rapid amplification of 5′ cDNA ends (5′-RACE) and 3′ cDNA ends (3′-RACE) was performed on mRNA using the GeneRacer kit (Invitrogen). PCR amplifications were performed using the GeneRacer primer and the nesting gene-specific primers (see Additional files 3 and 4) under the following conditions: predenaturation at 94°C for 2 min, 5 cycles of amplification at 94°C for 30 s and 72°C for 1 min, 5 cycles of amplification at 94°C for 30 s and 70°C for 1 min, 25 cycles of amplification at 94°C for 30 s, 56°C for 30 s and 72°C for 2 min, followed by a final extension at 72°C for 15 min. Nested PCR amplifications were carried out using the GeneRacer nested primer and the nested gene-specific primers (see Additional files 3 and 4) under the following conditions: predenaturation at 94°C for 2 min, 30 cycles of amplification at 94°C for 30 s, 58°C for 30 s and 72°C for 2 min, followed by a final extension at 72°C for 15 min. PCR products were purified, cloned and sequenced.

Based on the obtained 5′ and 3′ cDNA sequence, gene-specific forward and reverse primers (see Additional file 5) were designed for amplification of full-length *SmAGO* cDNA. PCR amplifications were performed under the following conditions: predenaturation at 94°C for 2 min, 30 cycles of amplification at 94°C for 30 s, 56°C for 30 s and 72°C for 3 min, followed by a final extension at 72°C for 15 min. PCR products were gel-purified, cloned and sequenced.

### Bioinformatic analysis and phylogenetic tree construction

The molecular weight (MW) and theoretical isoelectric point (p*I*) were predicted using the Compute pI/MW tool on the ExPASy server (http://web.expasy.org/compute_pi/). Intron/exon structures were analyzed manually based on genomic DNA sequences and the cloned cDNA sequences. Conserved domains were analyzed by search the deduced amino acid sequence of *SmAGOs* against the NCBI conserved domain (http://www.ncbi.nlm.nih.gov/Structure/cdd/wrpsb.cgi). The conserved residues were analyzed by alignment of amino acid sequences using DNAMAN and then checked manually. For phylogenetic tree construction, amino acid sequences of AGOs from *S. miltiorrhiza*, *Arabidopsis* and rice were first aligned using ClustalW version 1.83 and then constructed using MEGA version 4.0 by the neighbor-joining method with bootstrap to be 1000 replicates [55,56].

### Quantitative real-time reverse transcription-PCR (qRT-PCR)

Total RNA was extracted from plant tissues using the plant total RNA extraction kit (BioTeke) and then treated with RNase-free DNase (Promega) to remove genomic DNA contamination. RNA integrity was analyzed on a 1% agarose gel. RNA quantity was determined using a NanoDrop 2000C Spectrophotometer (Thermo Scientific). Reverse transcription was performed on 1 μg total RNA by 200 U Superscript III reverse transcriptase (Invitrogen) in a 20 μl volume. The resulting cDNA was diluted to 200 μl with sterile water. qPCR was carried out in triplicates using the BIO-RAD CFX system (Bio-Rad). Gene-specific primers were listed in Additional file 6. *SmUBQ10* was used as a reference as previously described [26]. PCR was carried out in a 20 μl volume containing 2 μl diluted cDNA, 250 nM forward primer, 250 nM reverse primer, and 1 × SYBR Premix Ex Taq II (TaKaRa) using the following conditions: predenaturation at 95°C for 30 s, 40 cycles of amplification at 95°C for 5 s, 60°C for 18 s and 72°C for 15 s. The results from gene-specific amplification were analyzed using the comparative Cq method which uses an arithmetic formula, 2-ΔΔCq, to achieve results for relative quantification [57]. Cq represents the threshold cycle.

The levels of miR168a/b and miR403 were analyzed using the miRNA-specific poly(T) adaptor RT-PCR method [40]. Briefly, 1 μg DNaseI-treated total RNA was polyadenylated using the Poly(A) Tailing kit (Ambion) as described previously [40] and then reverse-transcribed into single-strand cDNA. qRT-PCRs were performed in triplicates as described [58]. *S. miltiorrhiza* 5.8S rRNA was used as an endogenous reference. Primers used for miRNA quantification were listed in Additional file 7.

### Identification of *S. miltiorrhiza* miRNAs with perfect or near-perfect complementarity to *SmAGOs*

Known plant miRNA sequences were downloaded from miRBase (release 19, http://www.mirbase.org/) [35]. Target search of known plant miRNAs was performed against ten full-length *SmAGO* cDNA sequences using psRNATarget [36]. The maximum expectations of 3.5 and the target accessibility-allowed maximum energy to unpair the target site of 50 were applied. The identified plant miRNA sequences were then aligned with the current assembly of the *S. miltiorrhiza* genome (Chen et al., unpublished) using the SOAP2 program with no more than 2 mismatches allowed [37]. Hairpin structures were predicted using the mfold program [38]. Criteria described by [59] were applied to annotate *S. miltiorrhiza* miRNAs.

### Mapping of *SmAGO* cleavage sites

*SmAGO* cleavage sites were mapped using the modified RNA ligase-mediated rapid amplification of 5′ cDNAs method as described [39]. PCRs were carried out on mRNA isolated from *S. miltiorrhiza* roots and pooled samples containing flowers, leaves, stem and roots using the GeneRacer 5′ primer and the nesting gene-specific primers (see Additional file 8). Nested PCRs were performed using the GeneRacer 5′ nested primer and the nested gene-specific primers (see Additional file 8).

## Abbreviations

AGO: Argonaute; DCL: Dicer-like; dsRNA: double-stranded RNA; miRNA: microRNA; Mw: Molecular weight; ORF: Open reading frame; pI: Isoelectric point; pri-miRNA: Primary miRNA; qRT-PCR: quantitative real-time reverse transcription-PCR; RACE: RNA ligase-mediated rapid amplification of cDNA ends; RDR: RNA dependent RNA polymerase; RISC: RNA-induced silencing complex; siRNA: small interfering RNA; sRNA: small RNA; ssRMA: single-stranded RNA; ta-siRNA: trans-acting siRNA; TCM: Traditional Chinese medicine; UTR: Untranslated region.

## Competing interests

The authors declare that they have no competing interests.

## Authors’ contributions

FS analyzed the data, performed qRT-PCR and RACE, and participated in writing the manuscript. SL designed the experiment and wrote the manuscript. Both authors have read and approved the version of manuscript.

## Supplementary Material

Additional file 1 **Conserved domains in SmAGO proteins.** DUF1785, PAZ and PIWI domains are shown. Click here for file

Additional file 2 **Alignment of the deduced SmAGO amino acid sequences.** DUF1785, PAZ and PIWI domains are indicated by dotted, solid and broken lines. The conserved DDH/H798 or DDD/H798 residues are indicated by arrows. Click here for file

Additional file 3 **Primers used for 5′-RACE of *SmAGOs.*** Complete set of primers used for 5′-RACE of *SmAGOs*. Click here for file

Additional file 4 **Primers used for 3′-RACE of *SmAGOs.*** Complete set of primers used for 3′-RACE of *SmAGOs*. Click here for file

Additional file 5 **Primers used for amplification of full-length *SmAGOs.*** Complete set of primers used for amplification of full-length *SmAGOs*. Click here for file

Additional file 6 **Primers used for qRT-PCR.** Complete set of primers used for qRT-PCR. Click here for file

Additional file 7 **Primers used for miRNA quantification.** Complete set of primers used for miRNA quantification. Click here for file

Additional file 8 **Primers used for mapping of *SmAGO *cleavage sites.** Complete set of primers used for mapping of *SmAGO* cleavage sites. Click here for file
