# Supplementary figures and images for: Genome-wide identification, molecular cloning, expression profiling and posttranscriptional regulation analysis of the Argonaute gene family in Salvia miltiorrhiza, an emerging model medicinal plant
Source: BMC Genomics. 2013 Jul 29;14:512. doi: 10.1186/1471-2164-14-512 (PMC3750313; doi:10.1186/1471-2164-14-512)

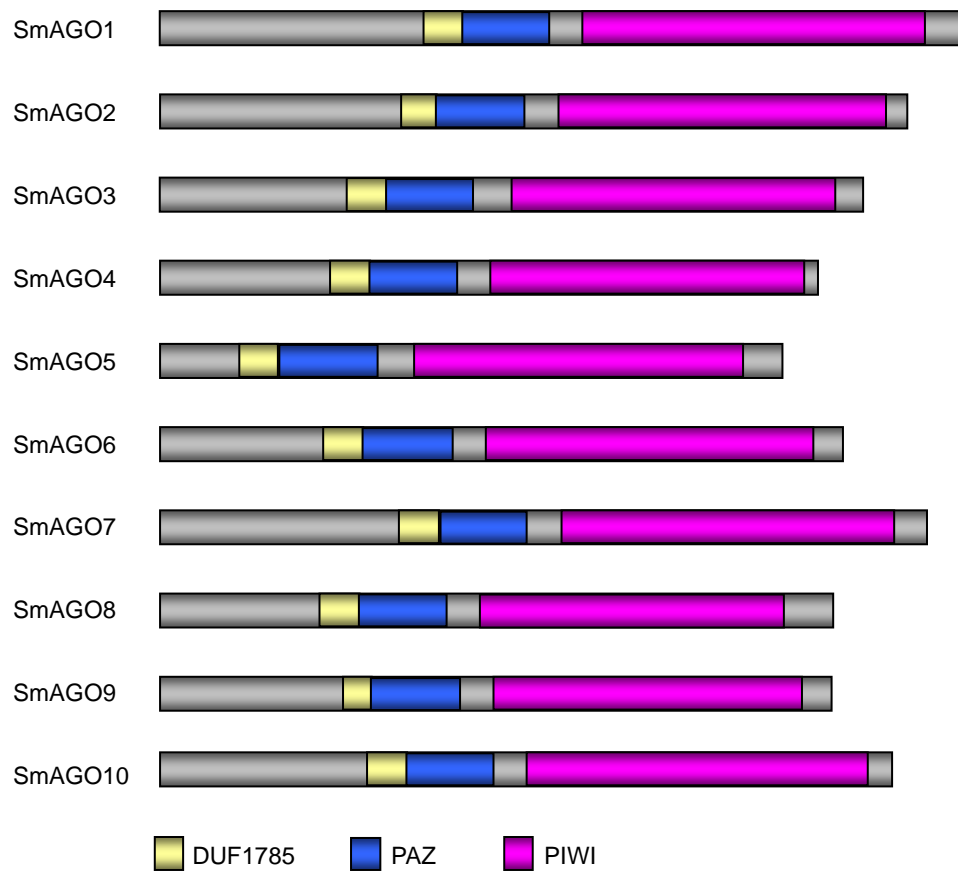

**Additional file 1.** Conserved domains in SmAGO proteins. DUF1785, PAZ and PIWI domains are shown.

Supplement: Additional file 1 — Conserved domains in SmAGO proteins. DUF1785, PAZ and PIWI domains are shown. [file 1471-2164-14-512-S1.pdf]
