# Supplementary material for: Genome-wide identification, molecular cloning, expression profiling and posttranscriptional regulation analysis of the Argonaute gene family in Salvia miltiorrhiza, an emerging model medicinal plant
Source: BMC Genomics. 2013 Jul 29;14:512. doi: 10.1186/1471-2164-14-512 (PMC3750313; doi:10.1186/1471-2164-14-512)
Supplement: Additional file 3 — Primers used for 5′-RACE of SmAGOs. Complete set of primers used for 5′-RACE of SmAGOs. [file 1471-2164-14-512-S3.pdf]

Additional file 3. Primers used for 5'-RACE of *SmAGOs*

**Gene name**	**Primer (5' to 3')**
SmAGO1	nesting: CCTGCGCAGACGCTTCCACCTAT
	nested: GTGGCAACTCCAGGCCTAGCCAT
SmAGO2	nesting: GTCCTTGTCTGGAAGCTCTGCAA
	nested: CTAGGCTTAGTCGTCACAGCTTGA
SmAGO3	nesting: GACAGGCCCATCTGAGTAGGTCTT
	nested: CTGCAGAGCTTCCCTCGGACCAT
SmAGO4	nesting: GCGAGGAAGTGAGCCAACAGTGA
	nested: GTTCCAAACCCACGTCTAGCCAT
SmAGO5	nesting: CGTGGAAGCGAACCAACAGTGAA
	nested: GTTCCAGGGCCACGTCTAGCCATT
SmAGO6	nesting: GTGGCGTGGATGCTCCTCAACAA
	nested: CCACCCCACCAGAATCTGGCCTT
SmAGO7	nesting: CTTGCCGTCAACAGCTGTATTGGT
	nested: GTCAGCAGACTCAGCACCTTCTA
SmAGO8	nesting: GAGGTTGATCTTCCGAACGGCAA
	nested: GAGGGTTGGGTTGCCTCGAAGATT
SmAGO9	nesting: GTATCAAACGCAGTCCAGCCTTGT
	nested: CCGCCTGACAGGTAAAATTCTGT
SmAGO10	nesting: CGTTGGCAGGTCGCCTTCAGCAA
	nested: GTATCACTGCCTGCCTGAACAGCT
