# Supplementary material for: Genome-wide identification, molecular cloning, expression profiling and posttranscriptional regulation analysis of the Argonaute gene family in Salvia miltiorrhiza, an emerging model medicinal plant
Source: BMC Genomics. 2013 Jul 29;14:512. doi: 10.1186/1471-2164-14-512 (PMC3750313; doi:10.1186/1471-2164-14-512)
Supplement: Additional file 4 — Primers used for 3′-RACE of SmAGOs. Complete set of primers used for 3′-RACE of SmAGOs. [file 1471-2164-14-512-S4.pdf]

Additional file 4. Primers used for 3’-RACE of *SmAGOs*

Gene name	Primer (5' to 3')
SmAGO1	nesting: CGATGAGCTCGGCTACTCTCCTGA
	nested: CTGTGTGAGAATGTCCGCAGCTCA
SmAGO2	nesting: CATGACAGGCATGCAATCTGCCAT
	nested: CTGTGAGGCCCCTACCACAGCTA
SmAGO3	nesting: GTACGCGAGGTGCACGCGTTCAGT
	nested: GGCACACGAGTAGCCGGAGAGTT
SmAGO4	nesting: GACGGCACAACGAGGCCTACTCAT
	nested: GTTGCAGGAGAGTGTGCGTAACT
SmAGO5	nesting: GCTGCGGTGGTTAGCTCAAGACA
	nested: CTCTGTACAAACGGCCAGACCAGA
SmAGO6	nesting: GACGGTCGTGGACACCGTCATCA
	nested: GCTGGTCTACAACCTCTGCTACA
SmAGO7	nesting: GCATTCCAATCGCATTCCACTAGT
	nested:GTTGGATCACGAAGCTGGCCATT
SmAGO8	nesting: GTCTCGCTTGTTCCACCTGTGTA
	nested: CTACGATCTGCACCCTGATCTGT
SmAGO9	nesting:GTCGCTTGTTCCGCCAGTTTACT
	nested: CTCCACCCTGATCTTCAGAACAT
SmAGO10	nesting: GACAGTGGATCGACTTCGGATGCT
	nested: GGACAATGTGAAGGACGTGATGT
