# Supplementary material for: Genome-wide identification, molecular cloning, expression profiling and posttranscriptional regulation analysis of the Argonaute gene family in Salvia miltiorrhiza, an emerging model medicinal plant
Source: BMC Genomics. 2013 Jul 29;14:512. doi: 10.1186/1471-2164-14-512 (PMC3750313; doi:10.1186/1471-2164-14-512)
Supplement: Additional file 5 — Primers used for amplification of full-length SmAGOs. Complete set of primers used for amplification of full-length SmAGOs. [file 1471-2164-14-512-S5.pdf]

Additional file 5. Primers used for amplification of full-length *SmAGOs*

**Gene name**	**Primer (5' to 3')**
SmAGO1	forward: GATCAGCAACTAGAGATGGACA
	reverse: CTGAGACATCTGGGCAGCAGCAA
SmAGO2	forward: CAAGCTGTGACGACTAAGCCTAGT
	reverse: CTTAGCTGTGGTAGGGGCCTCACA
SmAGO3	forward: GGTCCGAGGGAAGCTCTGCAGAT
	reverse: CTCTCCGGCTACTCGTGTGCCTT
SmAGO4	forward: GGCTAGACGTGGGTTTGGAACTA
	reverse: GGATGAAGTCTCCGACGCATCCT
SmAGO5	forward: GGCTAGACGTGGCCCTGGAACAA
	reverse: GGAGATTGAGTTCGAACAGCTGCT
SmAGO6	forward: GTTGTGACCCTTCTTGCCAACCAT
	reverse: GTAGCAGAGGTTGTAGACCAGCT
SmAGO7	forward: GGAAAACGTGTCTCTTTGCTTGCA
	reverse: CATCCACCAAAACCACATGACCAT
SmAGO8	forward: CTCCCACGAGTTCTGAGAGTGGTA
	reverse: GTGGAAGATGTTCCTCGAGGGTCT
SmAGO9	forward: CAGGCAATTAGTATCCCGTGGCA
	reverse: GGAACTCCTTGGCCGCTTCTTGA
SmAGO10	forward: GCTGTTCAGGCAGGCAGTGATACA
	reverse: CATCACGTCCTTCACATTGTCCT
