# Supplementary material for: Genome-wide identification, molecular cloning, expression profiling and posttranscriptional regulation analysis of the Argonaute gene family in Salvia miltiorrhiza, an emerging model medicinal plant
Source: BMC Genomics. 2013 Jul 29;14:512. doi: 10.1186/1471-2164-14-512 (PMC3750313; doi:10.1186/1471-2164-14-512)
Supplement: Additional file 6 — Primers used for qRT-PCR. Complete set of primers used for qRT-PCR. [file 1471-2164-14-512-S6.pdf]

Additional file 6. Primers used for qRT-PCR

Gene name	Primer (5' to 3')
SmUBQ10	forward: AGATGGGCGGACACTTGCTGATTA
	reverse: ACTCTCCACCTCCAAAGTGATGGT
SmAGO1	forward: GCAGGCAGCCTCGTTCAAAGACA
	reverse: GAAGGACCACTGTTGAGCAGCATT
SmAGO2	forward: GCAGAGCTTCCAGACAAGGACTT
	reverse: GGTAGTGGTCCAGCAGTGTACAGA
SmAGO3	forward: GTCCGAGGGAAGCTCTGCAGATT
	reverse: GACAGGCCCATCTGAGTAGGTCTT
SmAGO4	forward: CACTGTTGGCTCACTTCCTCGCA
	reverse: CACCGCCTCCAACATCAGCGAAGT
SmAGO5	forward: CACTGTTGGTTCGCTTCCACGTA
	reverse: CGCCTGCATAGGAATCTTAGCA
SmAGO6	forward: CTTCATCAGCCTCCCGATATCTGT
	reverse: CAGCACCACATCAAGAGCATGGA
SmAGO7	forward: CCGCTCCTTGTCTCTTGTGGTAT
	reverse: GACTAGAATCACAGCGTCCTGAGA
SmAGO8	forward: GATCAACCTCGTGGTCAACCACTT
	reverse: CTTCTCGCCGTCGTAGACTGTTCT
SmAGO9	forward: CTCTGCAGTCACTACGGGCGAATT
	reverse: CAGCATAGTAAACTGGCGGAACAA
SmAGO10	forward: GGAGTGCAATGCGCAACCCTTAGT
	reverse: GGTTGGCAACACTGTGAGACGATA
