# Supplementary material for: Genome-wide identification, molecular cloning, expression profiling and posttranscriptional regulation analysis of the Argonaute gene family in Salvia miltiorrhiza, an emerging model medicinal plant
Source: BMC Genomics. 2013 Jul 29;14:512. doi: 10.1186/1471-2164-14-512 (PMC3750313; doi:10.1186/1471-2164-14-512)
Supplement: Additional file 7 — Primers used for miRNA quantification. Complete set of primers used for miRNA quantification. [file 1471-2164-14-512-S7.pdf]

Additional file 7. Primers used for miRNA quantification.

Primer name	Primer sequence (5' to 3')
miR168a/b	TCGCTTGGTGCAGGTCGGGAA
miR403	TTAGATTCACGCACAAACTCG
5.8S rRNA	GCTCTCGCATCGATGAAGAACGTA
Poly(T) adapter	GCGAGCACAGAATTAATACGACTCACTATAGG(T)12VN*
Reverse primer	GCGAGCACAGAATTAATACGAC
