# Supplementary material for: Genome-wide identification, molecular cloning, expression profiling and posttranscriptional regulation analysis of the Argonaute gene family in Salvia miltiorrhiza, an emerging model medicinal plant
Source: BMC Genomics. 2013 Jul 29;14:512. doi: 10.1186/1471-2164-14-512 (PMC3750313; doi:10.1186/1471-2164-14-512)
Supplement: Additional file 8 — Primers used for mapping of SmAGO cleavage sites. Complete set of primers used for mapping of SmAGO cleavage sites. [file 1471-2164-14-512-S8.pdf]

Additional file 8. Primers used for mapping of *SmAGO* cleavage sites

Gene name/miRNA name	Primer (5' to 3')
SmAGO1/miR168	nesting: GCAAGTCCTTGTCTGGAAGCTCT
	nested: CACCACACACTTTGTGCCATA
SmAGO1/miR530	nesting: GCTGTCAACGATACGCTACGTAACG
	nested: CGCTACGTAACGGCATGACAGTG
SmAGO2/miR403	nesting: CTAGCAAACCAGAAAAGCAGCA
	nested: GGGGTATGCTTCCTGTGAGCAT
SmAGO3/miR396*	nesting: GTAGGTTGACTGATCTTCCGACA
	nested: CGGAGTTTTGTGGTGAGCTCAA
SmAGO5/miR169*	nesting: CTGGTTGAGGATGAGTAACGTCT
	nested: CACCGCAAGGTCCAATACGCTA
SmAGO8/miR167*	nesting: CACCTCCCAAGTCCACGTAGTT
	nested: CTGTCATCGTTGTGGAAGAAGGA
