# Supplementary material for: Genome-wide identification, molecular cloning, expression profiling and posttranscriptional regulation analysis of the Argonaute gene family in Salvia miltiorrhiza, an emerging model medicinal plant
Source: BMC Genomics. 2013 Jul 29;14:512. doi: 10.1186/1471-2164-14-512 (PMC3750313; doi:10.1186/1471-2164-14-512)
Supplement: Additional file 2 — Alignment of the deduced SmAGO amino acid sequences. DUF1785, PAZ and PIWI domains are indicated by dotted, solid and broken lines. The conserved DDH/H798 or DDD/H798 residues are indicated by arrows. [file 1471-2164-14-512-S2.pdf]

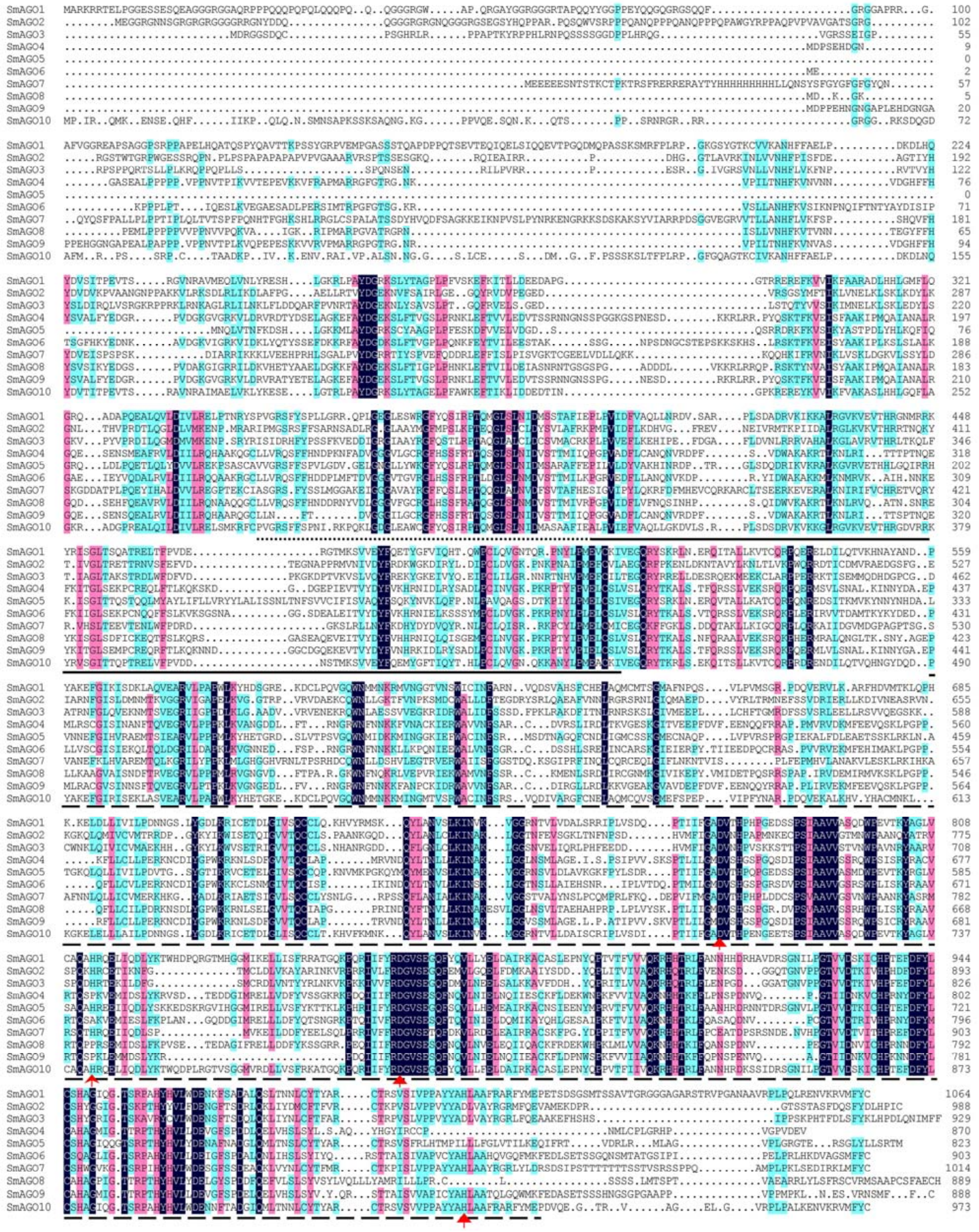

Additional file 2. Alignment of the deduced SmAGO amino acid sequences. DUF1785, PAZ and PIWI domains are indicated by dotted, solid and broken lines. The conserved DDH/H798 or DDD/H798 residues are indicated by arrows.
